# Packaging and Labeling of Pharmaceutical Products Obtained from the Internet

**DOI:** 10.2196/jmir.1441

**Published:** 2011-02-15

**Authors:** Michael Veronin

**Affiliations:** ^1^Rangel College of PharmacyDepartment of Pharmaceutical SciencesTexas A&M Health Science CenterKingsville TexasUnited States

**Keywords:** Internet pharmacy, online pharmacy, drug importation, drug label, pharmaceutical packaging

## Abstract

**Background:**

For patients, the prescription container label may be the only source of instructions on how to take their medicines. In the United States, the legal requirements for a prescription label are set by federal law and state statutes. The container should be comparable to that which manufacturers use to package drug products and should preserve a product’s identity, strength, quality, and purity and prevent contamination. Safety features such as a child-resistant closure should be provided. Pharmaceutical products purchased from international online pharmacies are not approved by the Food and Drug Administration (FDA) and may not meet US guidelines for labeling and packaging.

**Objective:**

The study objective was to determine whether commonly purchased pharmaceutical products obtained from international online pharmacies are comparable to products dispensed in the United States with regard to labeling and packaging.

**Methods:**

During March 2006 through January 2007, 41 pharmaceutical oral dosage form samples were obtained from international Internet pharmacy websites for evaluation: 18 generic simvastatin samples, 18 generic amlodipine samples, and 5 generic sildenafil samples. Contents for each package were observed and recorded and comparison of the prescription labeling and packaging of these products was made with prescription labeling and packaging requirements in the United States.

**Results:**

Of the 41 drug products obtained from online pharmacies from 12 different countries, only 1 product (from Canada) would meet both labeling and packaging guidelines for products dispensed in the United States. Of those not meeting the requirements, 7 were dispensed in paper envelopes with label affixed that was either handwritten or typed and contained missing information such as name and address of dispenser, name of prescriber, name of patient, and directions for use. Another 3 products did not have a label affixed to the drug product, but information was printed on a paper document enclosed in the shipping package, while 28 products did not have labels affixed to the drug product. In all, 39 of the 41 drug products’ packaging would not meet the US guidelines. Aside from the Canadian product, only 1 product from Mexico was dispensed in a container that would meet guidelines established in the United States. In total, 35 products were not dispensed in plastic vials but were dispensed in unit dose packages, paper envelopes with loose dosage forms, blister packs of drugs held together with rubber bands, or a combination of these packaging forms.

**Conclusions:**

Results suggest that labeling and packaging standards for international generic drug products are not equivalent to labeling and packaging standards in the United States. This suggests dissimilar and substandard distribution processes compared with those in the United States, which in turn presents a challenge to patient comprehension and health literacy and may affect patient adherence to drug treatment regimens. These findings have strong implications for drug product quality, patient outcomes, therapeutic effectiveness, and safety.

## Introduction

For patients, the prescription container label may be the only source of instructions on how to take their medicines [1]. According to the United States Pharmacopeia (USP),

the term “labeling” designates all labels and other written, printed, or graphic matter upon an immediate container of an article or upon, or in, any package or wrapper in which it is enclosed, except any outer shipping container. The term “label” designates that part of the labeling upon the immediate container [2].

In other words, “labeling” can refer to drug products from a manufacturer, and “label” can apply to drug products dispensed by a pharmacist on a prescription order.

In the United States, the legal requirements for a prescription label are set by federal law and state statutes [2,3]. At the federal level, the required items of information for the prescription product label can be found in Section 503 (b) (2) of the Federal Food, Drug, and Cosmetic Act (Table 1) [3]. States may have additional labeling requirements required by law [2].

**Table 1:** Guidelines for prescription labeling and packaging

Prescription Labels^a^	Prescription Packaging^b^
Serial number of prescription Date of prescription or date of its filling Name of prescriber Name of patient if stated on prescription Directions for use, including precautions, if contained in prescription	Light-resistant; Protects light-sensitive product and/or contents against photochemical deterioration Moisture-proof closure Child-resistant container with safety closure Container preserves product’s identity, strength, quality, purity as Type as specified by manufacturer

^a^ Federal requirements only; individual state requirements may differ

^b^ Includes requirements set by drug manufacturers dispensed to patients by licensed practitioners; exceptions include drugs requiring direct and immediate access (eg, oral contraceptives and certain cardiac drugs)

The National Association of Boards of Pharmacy (NABP) provides recommendations for state statutes on required items of information for outpatient prescription labels [4]. These recommendations augment federal requirements and include such items as name (proprietary or generic) and strength of drug product dispensed, special requirements with regard to the name of the product if an equivalent drug product is dispensed, name of the manufacturer or distributor of the product dispensed, beyond-use date of the product, quantity dispensed, number of refills, and name or initial of the dispensing pharmacist.

In the United States, it is the professional responsibility of pharmacists to label the dispensed drug product with the items stated in the federal law in addition to any state requirements for the state in which he or she is practicing.

In addition to proper labeling, pharmacists should select packaging that maintains the integrity of the drug product. The pharmacist’s choice of container should be based primarily on the type and quantity of medication to be dispensed and manner of use [5]. The container should be comparable to that which manufacturers use to package drug products and should preserve a product’s identity, strength, quality, and purity and prevent contamination. The type of container to be used by a pharmacist when dispensing a prescription drug is found in the manufacturer’s prescription product’s labeling and is regulated by the US Food and Drug Administration (FDA). The FDA regulation does not apply to products intended to be dispensed in the manufacturer’s original container. Manufacturer’s packaging and storage information is generally found on the original container or in the package insert.

In addition to packaging requirements for dispensing drug products, special consideration should be made for the closure on the prescription container. The closure must inhibit penetration of moisture and contaminants that can have a deleterious effect on oral dosage forms. A well-known example in pharmaceutics is the breakdown of aspirin into acetic acid and salicylic acid in the presence of moisture.

If the original package is intended to go directly from the pharmacist to the patient, manufacturers must place prescription drugs in child-resistant packages. Similarly, pharmacists must dispense prescription drugs for oral use to the patient in containers with child-resistant safety closures unless the patient or prescriber specifically requests otherwise.

A request for a safety container that is not child resistant must be obtained by the patient as a signed waiver and may apply to all of a patient’s dispensed medications. The pharmacist must maintain a record of the signed waiver request. Exceptions exist to these requirements, such as packaging of oral contraceptives because of their functional design, and certain types of cardiac drugs, such as nitroglycerin. In these instances, patients may need immediate access to the medication.

In addition, the closure must comply with guidelines specified in the Poison Prevention Packaging Act 1970 [6]. This federal law was enacted as a result of reports of a significant number of accidental poisonings of children after ingestion of household chemicals, including medications. Closures that must comply with these safety guidelines include both legend and over-the-counter (OTC) drug products.

Often, prescription drugs are sold in bottles with a seal that must be removed the first time the bottle is opened. This is an example of “tamper-evident” packaging that was introduced in the 1980s. The design of tamper-evident packaging makes it apparent if the packaging has been previously opened. The Tylenol crisis of 1982 highlighted the need for manufacturers to provide safeguards to altering drug product packaging [7].

In general, pharmaceutical products purchased from international online pharmacies are unapproved by the FDA, and in addition to possibly not meeting standards of formulation, may not meet quality standards for labeling and packaging [8]. International online pharmacies may offer convenience and potential cost savings to consumers, but potential health risks exist to patients from these types of drug purchases [9,10].

Currently, there is a paucity of information on packaging and labeling of pharmaceutical products imported via the Internet. The Office of Compliance in FDA’s Center for Drug Evaluation and Research initiated a study to determine the quality of a select group of pharmaceutical products purchased via the Internet from foreign sources. Packaging was a significant problem with virtually all of the Internet purchase samples. Many had either no or minimal labeling information for proper use. Some drug samples were shipped loosely in unlabeled plastic bags [11].

This paper reports on the variability of labeling and packaging for drug products obtained via the Internet. The objective of this study was to determine whether commonly purchased pharmaceutical products obtained from international online pharmacies are comparable to products dispensed in the United States with regard to labeling and packaging. This information is valuable for identifying trends in drug quality that may exist with consumer drug importation via the Internet.

## Methods

### Website Identification and Sample Acquisition

Drug product acquisition and website attributes were described previously in a report on drug quality, and a similar process was followed for this study [12]. For all other drug product samples, searches of the World Wide Web were conducted with the browser Internet Explorer 6.0. The websites for the Internet pharmacies were located by using the advanced search options of Google (http://www.google.com). The keywords selected for entry into the query box of the browser included the search terms “generic simvastatin,” “generic amlodipine,” generic sildenafil,” “online pharmacies,” and “Internet pharmacy.” All proprietary forms of oral drug products were identified on the websites for prospective procurement. Our perspective was that of a consumer seeking to purchase these prescription medications online or comparison price shopping. For Internet drug purchases, a consumer credit card was used for financial transactions as specified on the websites. Prescription requirements were noted, and for drug products requiring a prescription (ie, products obtained from Canada), a prescription was issued by a physician from the Texas Tech University Health Sciences Center School of Medicine and faxed to the Internet pharmacy as specified on the website.

### Physical Characterization

Upon receipt of drug product samples, the contents for each package were observed and recorded, and data were organized in tabular format. Comparison was made between the shipped items and prescription labeling and packaging requirements in the United States. Any additional distinguishing qualities were noted.

## Results

### Website Identification and Sample Acquisition

During March 2006 through January 2007, simvastatin, amlodipine, and sildenafil drug product samples were purchased from international markets from 41 websites. A checklist of attributes was created for each specific drug product (Table 2).

**Table 2:** Characteristics of pharmaceutical tablet samples from international markets obtained from the Internet

Product Name ^a^		Manufacturer	Lot Number	Product Expiration Date	Product Source (Laboratory)	Shipping Source	Product Source (website URL)
**Simvastatin**							
	Apo-Simvastatin	Apotex	GP5249	2006 AL	Toronto, Ontario, Canada	Canada	http://www.canadamednet.com^b^
	Co-Simvastatin	Cobalt	AC641	02-2006	Mississauga, Ontario, Canada	Canada	http://www.minitdrugs.com^b^
	Novo-Simvastatin	Novopharm	20392C	05-2006	Toronto, Ontario, Canada	Canada	http://www.Canadacure.com^b^
	Pms-Simvastatin	Pharmascience	20329A	01-2006	Montreal, Quebec, Canada	Canada	www.universaldrugstore.com^b^
	Simvastatin	Generics (United Kingdom) Limited	5C07SH	03-07	Hertfordshire, England, United Kingdom	United Kingdom	www.CanadaPharmacy.com^b^
	Simlo-20	Ipca Laboratories	VO4010R	11-07	Mumbai, Maharashtra, India	India	www.safemeds.com
	SIMLIP-20	Okasa Pharma	NS6002	12-07	Mumbai, Maharashtra, India	India	www.qualitygenerics.com
	Starstat 20	Mepro Pharm	03065M	05-07	Mumbai, Maharashtra, India	India	overseasrxdrugs.com
	Simi-20	Preet Pharm	PMI-2001	07-07	Delhi, India	India	www.fairrx.com
	Zorced	Productos Farmaceuticos Collins SA de CV	2734F4K	11-06	Guadalajara, Jalisco, Mexico	Mexico	www.pharmacymex.com
	Simvastatina ratiopharm	Ratiopharm Espana	Z-03	11-08	Madrid, Spain	Spain	www.tristatemeds.com
	Simlo-20	Ipca Laboratories	V05007R	05-07	Mumbai, Maharashtra India	India	www.xlpharmacy.com
	Simvastatin	Unicure Remedies	24205	05-07	Vadodara, Gujarat, India	India	www.worldremedium.com
	Simastin 20	Zaneka Healthcare Pvt	SAA602	12-07	Haridwar, India	India	www.generic-pharmacy-online.net
	Bestatin 20	Berlin Pharmaceutical Industry	06000065	01-09	Bangkok, Thailand	Thailand	www.healthworldconnect.com
	Simlup-20	Mepro Pharm	08124M	11-06	Mumbai, Maharashtra, India	India	www.rx2world.com
	Simlup-20	Mepro Pharm	02044M	03-06^c^	Mumbai, Maharashtra, India	Fiji Islands	www.inhousepharmacy.com
	Simvastatin	Not provided	None	03-08	Not provided	India	www.supersavermeds.com
**Amlodipine**							
	Amlodac-5	Cadila	ZF1141	03-08	Dholka, Ahmedabad, India	India	www.qualitygenerics.com
	Amlip-5	Okasa Pharma	NN6004	01-09	Mumbai, Maharashtra, India	India	www.freedoms-pharmacy.com
	Amlip-5	Okasa Pharma	NN5007	06-08	Mumbai, Maharashtra, India	India	www.rx-list.net
	Aginal-5	Alembic Ltd	6931003A	02-08	Vadodara Gujarat India	India	www.valuepharmaceuticals.com
	Norvasc	Pfizer New Zealand	55805036	10-10	Auckland, New Zealand (Made in China)	Fiji Islands	www.inhousepharmacy.com
	Amlopres-5	Cipla Ltd	Not provided	12-08	Mumbai Central, Mumbai, India	India	www.npmeds.com
	Amlopine	Berlin Pharmaceutical	0600652	04-09	Bangkok, Thailand	Thailand	www.1anabolic-steroids.com
	Amlodac-5	Cadila	ZF1143	03-08	Dholka, Ahmedabad, India	India	www.sharpmeds.com
	Amlibon	Ind-Swift, Ltd	AC5J02J	09-07	Samba, Jammu, India	India	www.xlpharmacy.com
	Amdepin-5	IRM Pharma	6001	02-08	Dholka, Ahmedabad, India	India	www.aclepsa.com
	Delfidin	Akums Drugs & Pharmaceuti-cals	06085AAV	07-07	Ranipur, Haridwar, India	Seychelles	www.budgetmedicines.com
	Elpress-5	Elder Pharmaceuti-cals	CSEL6001	03-09	TTC Industrial Area, Navi Mumbai, India	India	www.worldremedium.com
	Elpress-5	Elder Pharmaceuti-cals	CSEL 600308	07-09	TTC Industrial Area, Navi Mumbai, India	India	www.pharmacyforlife.com
	Norvas	Pfizer, SA de CV	6180501101	05-10	Toluca, Mexico	Mexico	www.pharmacymex.com
	Amlodipin	1A Pharma	6E0063	01-09	Oberhaching, Germany	Germany	www.tristatemeds.com
	Amlomed-5	Akums Drugs and Pharmaceuti-cals, Ltd	A1004	03-08	Ranipur, Haridwar, India	India	www.generic-pharmacy-online.net
	Amlokind-5	Unimax Labs	AD06-11	04-09	Faridabad, Haryana, India	India	www.pillsbasket.net
	Amlogard	Pfizer Ltd, India	620-05022	05-09	Navi Mumbai, India	India	www.discount-prescription-drugs-online.com
**Sildenafil**							
	Caverta	Not provided	Not provided	Not Provided	Not Provided	San Diego, California, United States	www.worldexpress.com
	Zenegra-100	Mepro Pharm	ZA 2002	02-03	Mumbai, Maharashtra, India	Monterrey, Neuvo Leon, Mexico	www.viagrasecrets.com
	Vega Asia	Not provided	B11102	10-04	Not provided	Delhi, India	www.blue-pills.net
	Suhagra-100	Okasa Ltd	MR3025	01-06	Pune, Maharashtra, India	India	www.viagrageneric.tripod.com
	Vega	Not provided	Not provided	04-04	Not provided	Manila, Phillippines	www.genericviagra.com

^a^ Product name as displayed on package at time of delivery

^b^ Prescription required as indicated by website

^c^ Expired at time of delivery

### Physical Characterization

Label and package characteristics of pharmaceutical products obtained from the Internet are presented in Table 3.

**Table 3:** Label and package characteristics of pharmaceutical products obtained from the Internet

Pharmaceutical Product Lot Number (Source)		Meets Label Standard^a^	Label Description	Meets Package Standard^a^	Package Description^b^
**Simvastatin**					
	GP5249 (Canada)	Yes	Standard prescription label for products dispensed in the United States	No	Round plastic vial: white, light-resistant, screw-top closure, not child resistant; original container from manufacturer
	AC641 (Canada)	Yes	Standard prescription label for products dispensed in the United States	No	Round plastic vial: white, light-resistant, screw-top closure, not child resistant; original container from manufacturer
	20392C (Canada)	Yes	Standard prescription label for products dispensed in the United States	No	Round plastic vial: white, light-resistant, screw-top closure, not child resistant; original container from manufacturer
	20329A (Canada)	Yes	Standard prescription label for products dispensed in the United States	Yes	Round plastic vial: white, light-resistant, screw-top child-resistant closure; original container from manufacturer
	5C07SH (United Kingdom)	No	Standard prescription label for products dispensed in the United States; missing name of prescriber	No	Unit dose strip packages taped together; blister pack with foil backing, not light resistant or childproof; original container from manufacturer
	VO4010R (India)	No	No label affixed to drug product; label information printed on enclosed paper; missing address of dispenser and name of prescriber	No	Unit dose strip packages enclosed in envelope; sealed foil pack, light resistant, not childproof; original container from manufacturer
	NS6002 (India)	No	No label affixed to drug product	No	Unit dose strip packs held together with rubber band; blister pack with foil backing, not light resistant or childproof; original container from manufacturer
	03065M (India)	No	No label affixed to drug product	No	Unit dose packages taped together; blister pack with foil backing, not light resistant or childproof; original container from manufacturer
	PMI-2001	No	Label affixed to envelope; missing name and address of dispenser, name of prescriber, name of patient, and directions for use	No	Unit dose strip packages enclosed in envelope; sealed foil pack, light resistant, not childproof; original container from manufacturer
	2734F4K (Mexico)	No	No label affixed to drug product	Yes	Round plastic vial: white, light-resistant, screw-top child-resistant closures; original container from manufacturer
	Z-03 (Spain)	No	No label affixed to drug product	No	Unit dose packages in box; blister pack with foil backing, not light resistant or childproof; original container from manufacturer
	V05007R (India)	No	No label affixed to drug product; label information printed on enclosed paper; missing address of dispenser and name of prescriber	No	Unit dose strip packages enclosed in envelope; sealed foil pack, light resistant, not childproof; original container from manufacturer
	24205 (India)	No	No label affixed to drug product	No	Unit dose strip packs held together with staples; blister pack with foil backing, not light resistant or childproof; original container from manufacturer
	SAA602 (India)	No	No label affixed to drug product	No	Unit dose boxes with strip packages enclosed in envelope; sealed foil pack, light resistant, not childproof; original container from manufacturer

	06000065 (Thailand)	No	No label affixed to drug product	No	Unit dose strip packs in envelope; blister pack with foil backing, not light resistant or childproof; original container from manufacturer
	08124M (India)	No	No label affixed to drug product	No	Unit dose strip packs in envelope; blister pack with foil backing, not light resistant or childproof; original container from manufacturer
	02044M (India)	No	No label affixed to drug product	No	Unit dose strip packs held together with rubber band; blister pack with foil backing, not light resistant or childproof; original container from manufacturer
	Lot No. not provided (India)	No	Nonstandard label affixed to drug product contains drug name and strength	No	Capsules placed in plastic container, not light resistant or childproof
**Amlodipine**					
	ZF1141 (India)	No	No label affixed to drug product	No	Unit dose strip packs held together with staples; blister pack with foil backing not light resistant or childproof; original container from manufacturer
	NN6004 (India)	No	Label affixed to envelope; missing name and address of dispenser, name of prescriber, name of patient, and directions for use	No	Unit dose boxes with strip packages enclosed in envelope; sealed foil pack, light resistant, not childproof; original container from manufacturer
	NN5007 (India)	No	Label affixed to envelope; missing name and address of dispenser, name of prescriber, name of patient, and directions for use	No	Unit dose boxes with strip packages enclosed in envelope; sealed foil pack, light resistant, not childproof; original container from manufacturer
	6931003A (India)	No	No label affixed to drug product	No	Unit dose strip packs in envelope; blister pack with foil backing, not light resistant or childproof; original container from manufacturer
	55805036 (New Zealand)	No	No label affixed to drug product	No	Unit dose packages in box; blister pack with foil backing, not light resistant or childproof; original container from manufacturer
	Lot number not provided (India)	No	No label affixed to drug product	No	Unit-dose strip packs enclosed in plastic wrap and placed in envelope; blister pack with foil backing, not light resistant or childproof; original container from manufacturer
	0600652 (Thailand)	No	No label affixed to drug product	No	Unit dose boxes with strip packs enclosed in envelope; blister pack with foil backing, sealed, light resistant, not childproof; original container from manufacturer
	ZF1143 (India)	No	No label affixed to drug product; label information printed on enclosed paper; missing address of dispenser and name of prescriber	No	Unit dose boxes with strip packs enclosed in envelope; blister pack with foil backing, sealed, light resistant, not childproof; original container from manufacturer
	AC5J02J (India)	No	No label affixed to drug product	No	Unit dose strip packs in envelope; blister pack with foil backing, not light resistant or childproof; original container from manufacturer
	6001 (India)	No	No label affixed to drug product	No	Unit dose strip packs in envelope; blister pack with foil backing, not light resistant or childproof; original container from manufacturer
	06085AAV (India)	No	No label affixed to drug product	No	Unit dose strip packs in envelope; blister pack with foil backing, not light resistant or childproof; original container from manufacturer
	CSEL6001 (India)	No	No label affixed to drug product	No	Unit dose strip packs in envelope; blister pack with foil backing, not light resistant or childproof; original container from manufacturer
	CSEL600308 (India)	No	Label affixed to envelope; missing name and address of dispenser, name of prescriber, name of patient, and directions for use	No	Unit dose strip packs in envelope; blister pack with foil backing, not light resistant or childproof; original container from manufacturer
	6180501101 (Mexico)	No	No label affixed to drug product	No	Unit dose boxes with strip packages enclosed in envelope; blister pack with foil backing, sealed, light resistant, not childproof; original container from manufacturer
	6E0063 (Germany)	No	No label affixed to drug product	No	One unit dose box with strip packages enclosed in envelope; blister pack with foil backing, sealed, light resistant, not childproof; original container from manufacturer
	A1004 (India)	No	No label affixed to drug product	No	Unit dose strip packs in loose plastic; blister pack with foil backing not light resistant or childproof; original container from manufacturer
	AD06-11 (India)	No	No label affixed to drug product	No	Unit dose strip packs wrapped in newspaper; blister pack with foil backing, not light resistant or childproof (front and back); original container from manufacturer
	620-05022 (India)	No	No label affixed to drug product	No	Two unit dose boxes with strip packages enclosed in shipping box; blister pack with foil backing, sealed, light resistant, not childproof; original container from manufacturer
**Sildenafil**					
	Lot number not provided (source not specified)	No	Label affixed to envelope; only contains drug name, strength, and directions	No	Loose tablets placed in small paper envelope enclosed in bubble wrap; container not light resistant, childproof, or moisture resistant
	ZA 2002 (India)	No	No label affixed to drug product	No	Unit dose strip pack in envelope; blister pack with foil backing not light resistant or childproof; original container from manufacturer
	B11102 (India)	No	Label affixed to envelope; only contains drug name and strength	No	Loose tablets placed in small foil unsealed envelope placed in bubble wrap envelope; container not light resistant, childproof, or moisture resistant
	MR3025 (India)	No	No label affixed to drug product	No	Twenty-four unit dose boxes held together with plastic tape; blister pack with foil backing, not light resistant or childproof; original container from manufacturer
	Lot number not provided (source not provided)	No	Handwritten label affixed to drug product; only contains drug name and expiration date	No	Loose tablets placed in small plastic bag; container not light resistant, childproof, or moisture resistant

^a^ Required items for dispensing to patients in the United States

^b^ Type of container dispensed to patients

Deviation from US federal requirements for prescription labels and manufacturer requirements for prescription packaging are noted. Among all samples obtained, significant variation was observed in external package appearance, labeling of drug product (if any), and packaging of dispensed product.

Of the 41 drug products obtained, only 1 product from Canada would meet both labeling and packaging guidelines for products dispensed in the United States. In all, 4 products from Canada were labeled in a manner that would meet US guidelines, and although the Canadian products were packaged in similar containers to those dispensed in the United States, 3 of 4 containers were not child resistant, and no offer was made to patients to dispense in this manner on the websites. A total of 7 products were dispensed in paper envelopes with a label affixed that was either handwritten or typed and contained missing information such as name and address of dispenser, name of prescriber, name of patient, and directions for use. Another 3 products did not have a label affixed to the drug product, but information was printed on a paper document enclosed in the shipping package. In all, 28 products did not have labels affixed to the drug product, while 39 of the 41 drug products’ packaging would not meet guidelines established in the United States. Aside from the Canadian product, only 1 product from Mexico was dispensed in a container that woul meet US guidelines. Of the 41 products, 35 were not dispensed in plastic vials but were dispensed in unit dose packages, paper envelopes with loose dosage forms, blister packs of drugs held together with rubber bands or staples, or a combination of these packaging forms. 

## Discussion

### Principal Results

**Figure 1:**
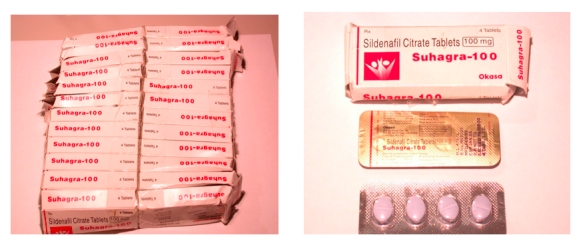
Generic sildenafil tablet obtained via the Internet without prescription

Results of this study indicate that drug products sold on the Internet—often accessible to consumers without prescription—present insufficient labeling and packing characteristics compared with products dispensed in the United States For example, picutred in Figure 1 is a tablet formulation of generic sildenafil (“Suhagra-100”) obtained from URL: http://www.viagrageneric.tripod.com. The tablets are ordinary in appearance, scored, contain no external markings with color variation from the US innovator product. Contents were packaged in unit dose plastic with foil backing, and the product appears to be from a legitimate drug manufacturer with manufacturer’s labeling. The shipping source was India as indicated from the international postal service packaging.

Of the 41 drug products, 36 did not meet criteria for prescription drug labeling, and 39 did not meet criteria for prescription drug containers and packaging as required in the United States. For all international products, the only drug products that met US standards for labeling were those from Canada. Only 1 container from Canada and 1 container from Mexico met US guidelines. No relationship could be ascertained between packaging standards and prescription requirements. 

### Comparison With Prior Work

Our findings corroborate a report by Westenberger et al, where drug sample packaging was a significant concern for virtually all of the Internet purchased samples [11]. In this report, several samples had either no or minimal labeling information for proper usage or testing of these drugs. For instance, some samples had packaging with labeling in foreign languages, and others were shipped loosely in unlabeled plastic bags.

### Clinical Implications

Medication errors have been attributed to improper labeling and packaging of medications. A major report from the Institute of Medicine (IOM) noted that problems with prescription drug labeling were cited as the cause of a large number of outpatient medication errors and adverse drug events (ADEs) [1]. In addition, the United States Pharmacopeia (USP) found that 33% of the reports to its voluntary Medication Errors Reporting (MER) database cited labeling or packaging as having contributed to a medication error, including almost 30% of the fatalities reported [13]. Some of the more common sources of medication errors are confusion between soundalike medication names or look-alike medication names and confusion due to similar appearances for medication packages or similar labels for different medications.

Patients may unintentionally misuse a prescribed medicine because of misunderstanding of instructions. Individuals who manage complex medication regimens were found to be at greater risk for making errors in interpreting container label instructions, particularly the elderly with limited literacy skills. The patient’s ability to understand prescription label instructions can be critical to safe use of medications since other sources of information on medicines for patients may not be adequate and pharmacists may not always have the opportunity to provide counseling to patients on prescribed medications [14].

Research in health literacy underscores the high prevalence of misunderstanding of seemingly simple instructions and warnings placed on prescription container labels by patients. Studies have demonstrated that the literacy level of patients has an impact on their ability to understand directions on a label [15]. Health literacy has increasingly been viewed as a patient safety issue, and lower literacy may contribute to medication errors [16]. Lower literacy and a greater number of prescription medications were independently associated with misunderstanding the instructions on prescription medication labels [17]. Prescription drug labels should use explicit dosing intervals and clear and simple language within a patient-friendly label format—unlike the drug products received from the Internet as demonstrated in this study. Although health literacy levels of Internet drug buyers is largely unknown, it is reasonable to assume that they are not dissimilar to all patients in the population.

In addition to literacy and drug product labeling, of equal importance are the drug product’s container and packaging, not only to preserve the product’s integrity, but to provide ease of use for both pharmacist and patient [18]. It has been demonstrated that patients actually prefer types of packaging and labeling that are designed for safety, are easier to read, and have better organized warnings with larger type size [16].

Studies suggest that certain patient groups such as the elderly may benefit from specialized packaging for drug products [18]. Providing medications in a package that identifies the day each dose is intended to be taken and provides information on proper self-administration can improve treatment regimen adherence and treatment outcomes in elderly patients. Certain patient groups may need the most attention to their packaging needs, yet this study suggests that these needs are unlikely to be met if drugs are purchased via the Internet.

Drugs obtained from international markets via the Internet can present a health risk to patients for a variety of reasons [19]. This study has demonstrated that it is highly likely that the average US consumer may obtain an imported drug product from an Internet pharmacy website that does not meet quality specifications of packaging and labeling equivalent to US-dispensed products—another potential safety risk.

In the United States, policies and legislation are directed toward ensuring minimal health risk to patients in the use of prescription drugs, as reported by the US Food and Drug Administration [8]. This report illustrates that consumers can obtain prescription drugs via the Internet without direct oversight from a health care professional. It has been stated that this ability to purchase medications directly from a website poses the greatest risk of drug adverse effects by bypassing the traditional “visit to the physician and a review by a pharmacist” [20], and this observation may be expanded to included issues of packaging and labeling. Consumers who order drugs from the Internet do not have sufficient access to information and advice at the point of ordering and on delivery to make informed decisions about their safe and appropriate use [21]. Again, substandard packaging and labeling may compound this risk.

This study has shown that a variety of packaging and labeling exists for pharmaceuticals obtained via the Internet. Based on the findings of this study, Canadian drug products dispensed are in many respects similar if not identical to US products, with similar prescription requirements [22]. Perhaps further distinction should be made by health care authorities between drugs imported from international markets in general and from Canadian manufacturers.

### Limitations

The relatively small sample size in this study may not be wholly representative of all drugs sold on the Internet and generalizing our findings to other drugs should be done with a degree of caution. However, our findings corroborate previously published work, and further studies are warranted to identify trends in quality for Internet drug product labeling and packaging.

A goal of this study was to assess quality attributes that may indirectly convey information on safety and effectiveness through packaging and labeling properties. Many factors contribute to quality of packaging and labeling and although significant, the list of attributes observed in this study was not exhaustive. Until a direct association between packaging and labeling of Internet drug products and clinical outcomes is established, again, one must interpret these findings with some reservation.

### Conclusions

Our findings indicate nonequivalent labeling and packaging for drug products available to consumers via the Internet compared with prescription drug products sold in the United States. These findings suggest dissimilar and substandard distribution processes compared with the United States, which in turn offer a greater challenge to patient comprehension and health literacy and may affect patient adherence to drug treatment regimens. In the United States, consumers need to be aware that, irrespective of advertising claims on Internet pharmacy websites, consumers may receive a drug product that is not equivalent to the US. counterpart and that may be dissimilar to products that would be allowed for consumers in the United States. These findings have strong implications for drug product quality, patient outcomes, therapeutic effectiveness, and safety that should be considered by clinicians to potentially safeguard patients who choose to purchase foreign-produced drugs via the Internet.

## Abbreviations

ADE: adverse drug event
FDA: Food and Drug Administration
IOM: Institute of Medicine
MER: Medication Errors Reporting
NABP: National Association of Boards of Pharmacy
OTC: over the counter
USP: United States Pharmacopeia
